# A hybrid zero-reference and dehazing network for joint low-light underground image enhancement

**DOI:** 10.1038/s41598-025-95366-3

**Published:** 2025-03-24

**Authors:** Qing Du, Shihao Zhang, Zhipeng Wang, Jincheng Liang, Shijiao Yang

**Affiliations:** School of Resources Environment and Safety Engineering, University of South China, Hengyang, 421001 Hunan China

**Keywords:** Underground mine, Zero-shot learning, Low-light enhancement, Image dehazing, Other photonics, Engineering, Mathematics and computing, Optics and photonics

## Abstract

The raw images captured by underground vision sensors in underground mine settings are disturbed by dim lighting, high dust levels, and complex electromagnetic conditions, suffering from high noise, low illumination, and low-resolution contamination, which further affects the supervision of the vision sensors. However, existing image enhancement methods relying on synthesized datasets are not suitable for improving images in real underground mine settings. This study focuses on addressing these challenges. We collected a large number of underground mine images and proposed a novel image enhancement approach. Inspired by visual image processing techniques, this approach combines low-light enhancement and dehazing methods to address the issues of uneven lighting and fog distortion. Specifically, the proposed Zero-Reference Depth Curve Estimation-Dehazing Network (Z-DCE-DNet) aims to enhance underground mine images. It addresses two key aspects: (1) enhancing low-light images by incorporating higher-order loss curves into the DCE-Net backbone and introducing a new loss function to optimize network learning for improved low-light image quality; (2) addressing the color distortion and blur caused by low light enhancement through post-processing using convolutional neural networks, with AOD-Net enhancing the clarity of downhole images. Extensive experimental results demonstrate that the Z-DCE-DNet method produces visually superior enhanced images, and comparative analyses of multiple object detectors reveal that the enhanced images lead to improved detection outcomes.

In underground mines, poor lighting conditions often lead to insufficient illumination in images captured by camera sensors, degrading the video quality. Additionally, the aesthetic appeal of images captured by underground vision devices is compromised, and the information transmission is unsatisfactory. Therefore, image enhancement in the context of underground mining should be a subject of research. It can effectively improve the visibility and visual quality of underground mining scenes. Moreover, enhanced visibility emphasizes scenes and objects more prominently, providing a stronger foundation for advanced computer vision tasks(e.g^1–4^). in mining, such as recognizing personnel behavior and detecting restricted areas.

Low-light enhancement is the primary task of underground mining images, which is a significant research direction in image processing. The latest advancements in low-light enhancement are mainly based on deep learning solutions^5^, incorporating various learning strategies such as supervised learning, reinforcement learning, unsupervised learning, zero-shot learning, and semi-supervised learning, along with training datasets(e.g^6–10^). These methods provide effective benchmarks for mining image enhancement. Supervised learning(e.g^11–14^). , reinforcement learning(e.g^15–19^). , and unsupervised learning all require the use of paired training datasets^20^. This requirement poses challenges in acquiring underground images that accurately depict the real scenes captured by mining visual equipment. Concerning generalization, these methods exhibit limited generalization capabilities and unstable training.

In addition, image enhancement models trained on synthetic images typically do not generalize well to real-world images. Many enhancement methods based on unsupervised learning aim to overcome the limitations of training on synthetic data. These methods take low-light images as input and generate high-quality curves as output to enhance the images. However, the curves used in this method may reduce image contrast and surface color, resulting in a blurry final image. Existing methods applied in underground mines still require further improvement.

To address these issues, we propose a lightweight method, named Z-DCE-DNet, for enhancing mine images that meets the practical requirements of mining operations. The Z-DCE-DNet framework consists of two stages: unsupervised low-light enhancement operation and dehazing sharpening processing. In the low-light enhancement stage, we use realistically captured underground images for unsupervised training, relying on the DCE-Net backbone. By using a carefully designed loss function, we can obtain the most suitable image enhancement parameters. Additionally, in the context of underground image enhancement, we retrain the images restored with illumination and then use a series of lightweight CNN network frameworks for dehazing, based on a physical scattering model. The effectiveness of our method is validated through multiple comprehensive sets of experiments.

To obtain clearer images of mines, we combine two image enhancement strategies: weak light enhancement^6^and image dehazing^7^, for image processing tasks in underground mine. The main contributions of this paper are as follows:

- **Images of Underground Mines Dataset (IUMD)**: Due to the lack of underground images in real-life scenarios. With the varying exposure levels in underground environments, we gathered surveillance video data from diverse underground scenes to create a more extensive dataset.
- **Unsupervised Image Enhancement Strategies**: To enhance the illumination of underground images, we improved the unsupervised learning method of DCE-Net. After enhancing the image, we revisited the color deviation issue and achieved improved image illumination by optimizing the non-reference loss function. Subsequently, to address the image blurriness caused by low light enhancement, we proposed a post-processing method based on AOD-Net to obtain clearer images.
- **Comparison Experiment of Image Enhancement Methods**: An ablative experiment was conducted to validate the effectiveness of the proposed method. Additionally, we analyzed and compared Z-DCE-D with current mainstream image enhancement methods. The experimental results indicate that our method outperforms other models in terms of subjective visual quality.
- **Comparison of Object Detectors**: To validate the benefits of enhanced images for subsequent visual tasks underground, we employed the current popular object detection algorithm. The performance of the safety helmet detection model in images was compared before and after enhancement. Results from multiple experimental comparisons showed that the enhanced images could further improve the performance of the object detection model.

The structure of this article is as follows: Sect. 2 introduces our dataset and provides a review and summary of the relevant literature. Section 3 delves into the details of the proposed framework. Performance evaluation is presented in Sect. 4. The article concludes with a summary in Sect. 5.

## Related work

In this section, we will first introduce our dataset and then summarize the most common image enhancement and image defogging methods. We reviewed the existing methods for multiscale feature representation and compared them with our proposed method.

### Images of underground mines dataset, IUMD

Low-light image enhancement (LLIE) aims to improve the perception or interpretability of an image captured in a low-light environment^21^. Supervised learning-based image recovery and enhancement methods rely heavily on paired datasets of synthesized low-light and normal images, such as LOL^18^, Exclusive Dark^22^, SID^9^, and RELLISUR^23^ among others.

Typically, pairing data is captured by automatically dimming lights, changing camera settings or retouching synthetic data. The data has increased difficulties when used in underground mine scenarios: (1) Capturing images of the same visual scene at the same time in an underground mine is difficult. (2) Synthesising damaged images from clean images can sometimes be helpful, but such synthesis results are usually not photorealistic enough, resulting in various artifacts when the trained model is applied to real low-light images.

**Fig. 1:**
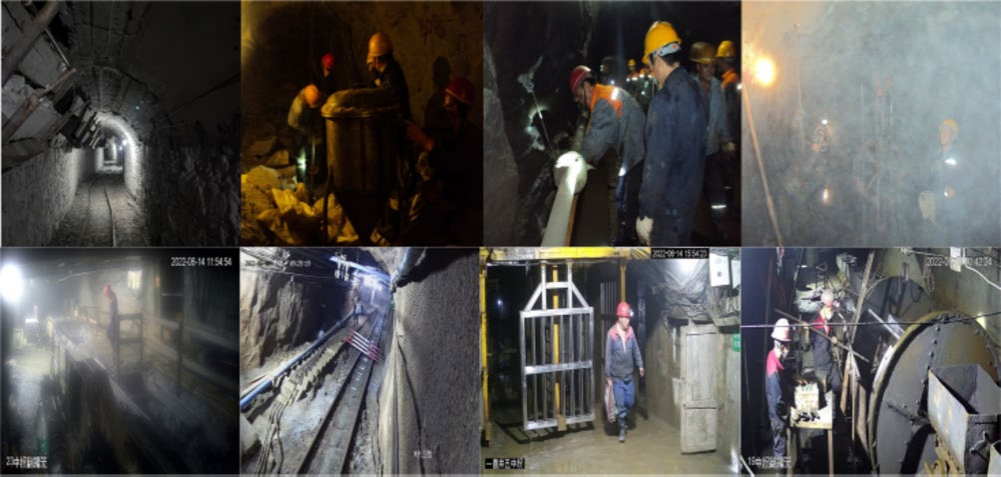
Uneven lighting, exposure of a single light source, blurriness, occlusion of complex scenes, etc., are all present in some of IUMD’s images, including downhole surveillance video and field images. The subsequent intelligent detection of these unprocessed images is severely hampered.

To do this, we set up the IUMD. We collected the actual surveillance video from 12 mines control rooms in Hunan, Shandong, and Guangdong provinces in China, as well as the images taken in the field under different light intensities. The section of the IUMD is shown in Fig. 1. There are several complex scenes like pit stop and track, a total of 1647 images under different lighting conditions, and the image size was set at 512 × 512. All volunteers who participated in photo documentation were informed about the data usage and agreed to the research content presented in this manuscript.

### Low light enhancement

Paired training data sets are required in volume for learning strategies, which presents great difficulties in the acquisition of subsurface images and may not reflect the real scene obtained by mine vision equipment. However, a zero-shot-based learning strategy is used to emphasize that this approach does not require paired or unpaired training data.

ExCNet is the first zero-shot CNN backlight image restoration method^24^. Through training and testing, the S-curve of the backlit image can be estimated images. This scheme can be applied to most backlight shooting scenes and backlight image restoration processing. RRDNet enhances the three components of light, emission rate^25^, and noise of low-quality images by combining retinal reconstruction loss, texture enhancement loss, and light-guided noise estimation loss. This method does not require pre-trained image samples or prior training and can be used to restore underexposed images. Zero-DCE formulates low-light enhancement as a task of image-specific curve estimation, which takes low-light images as input and produces higher-order curves as output^26^. These curves are adjusted to take into account the pixel value range, monotonicity, and differentiability of the input image to obtain an enhanced image, based on which Zero-DCE + + is proposed as a lighter version^27^.

### Image dehazing

Although the captured scene images are illuminated, problems such as low contrast, low saturation, loss of detail and color deviation after illumination are more severe in underground mines. Post-processing methods to improve image defogging are considered. Traditional image dehazing methods are mainly based on dark channel prior^28^ and color attenuation prior^29^. Better environmental conditions are required to apply the above methods. Deep learning-based methods are more flexible. MS CNN trained the network to transfer the matrix in a coarse-to-fine way to estimate haze, which is one of the earliest studies to solve the haze removal problem by CNN^30^. DehazeNet is a trainable end-to-end system that explicitly learns the mapping relationships between raw haze images^31^. DCPDN embedded the physical scattering model into the network, allowing the network to jointly estimate transmission graphs, atmospheric light, and haze removal images^32^. AOD-Net was designed to produce recovered images by reformulating the physical scattering model, and the same idea was later extended to video deblurring^33^. PSD is a principled composite to the real dephasing framework that starts from the dephasing model backbone of pre-trained synthetic data and uses real fuzzy images to fine-tune the model in an unsupervised manner^23^. The adjustment is made through experience to obtain a clearer image.

Image low-light enhancement and dehazing methods are crucial in image processing, and these techniques have demonstrated significant effectiveness in real-world scenarios. Inspired by previous work, we combine these two image processing methods, employing dehazing enhancement after low-light enhancement strategy to obtain clearer underwater images.

## Method

**Fig. 2:**
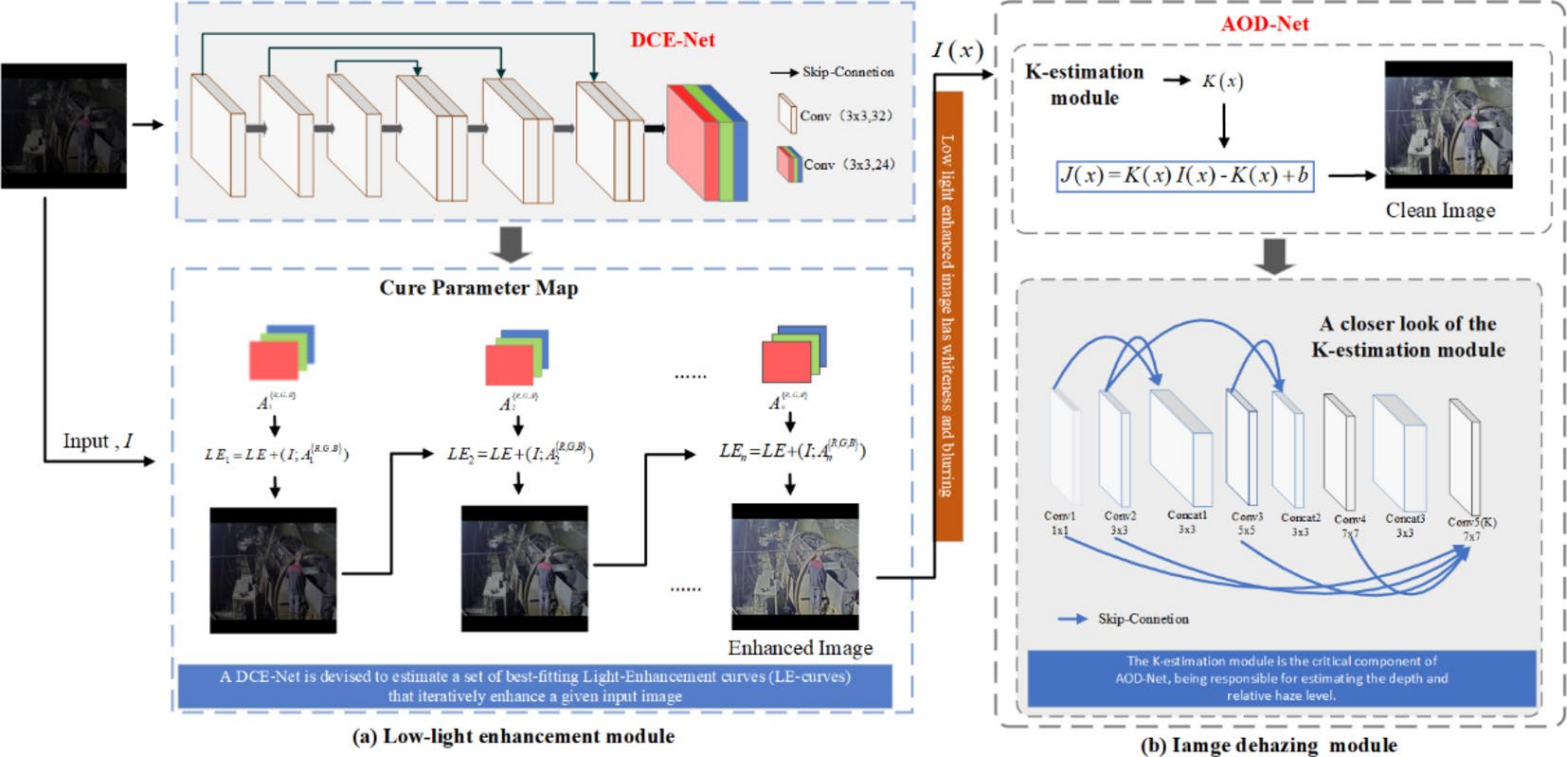
The framework of Z-DCE-DNet includes (**a**) an image low-light enhancement module and (**b**) a dehazing enhancement module. The detailed structure of the network is described later.

This section provides an in-depth discussion of the underground image enhancement method proposed in Z-DCE-DNet. As shown in Fig. 2, the complete proposed algorithm is summarized in two stages. In the first stage, we enhance the weak light in the images captured in underground mines. The second stage involves sharpening the images enhanced for luminance. Finally, a clearer image has been obtained. The detailed steps of each stage are elaborated in the following sections.

### Framework overview

The overall framework of the Z-DCE-DNet is shown in Fig. 2, which includes two stages of image enhancement for low light conditions and image dehazing.

(1) Low light enhancement module (Fig. 2-a): The backbone of the system is DCE-Net^26^, a deep network for zero-shot curve estimation. The input to DCE-Net is a set of low-light images acquired downhole, which are iteratively enhanced for a given input image (i.e., the enhanced image is used as the input for the next iteration, and that input is incrementally enhanced). The output is a set of pixelated curve parameter plots corresponding to higher-order curves that are iteratively adjusted to obtain the best brightness enhancement image.

(2) Image dehazing module (Fig. 2-b): The mine image processed only by the low-light enhancement method pays more attention to enhancing the brightness of the image, resulting in a loss of clarity of the brightened image. Inspired by the strong physical background of dehazing, the AOD-Net is used to deblurring. Estimate K(x) from the input fuzzy image$\:I\left(x\right)$, and then use $\:K\left(x\right)$ as the input parameter to estimate $\:J\left(x\right)$, that is, generate a clean image. The detailed network architecture is shown in Fig. 2.

### Low light enhancement module

DCE-Net is designed to learn the mapping between borehole images and their best-fit curve parameter mappings, using a normal CNN with seven convolutional layers with symmetric cascades^26^. Each layer consists of 32 3 × 3 convolutional kernels with a step size of 1, followed by the ReLU activation function. The last convolution layer produces 24 parameter graphs for 8 iterations (*n* = 8), where each iteration requires three curve parameter graphs for the three channels R, G & B.

A distinguishable set of non-reference losses is proposed in Zero-DCE, which enables us to evaluate the quality of enhanced images^26^. The following four types of losses are used to train DCE-Net: (1) Spatial Consistency Loss. (2) Exposure Control Loss; (3) Color constancy loss; (4) Illumination smoothness Loss.

**Fig. 3:**
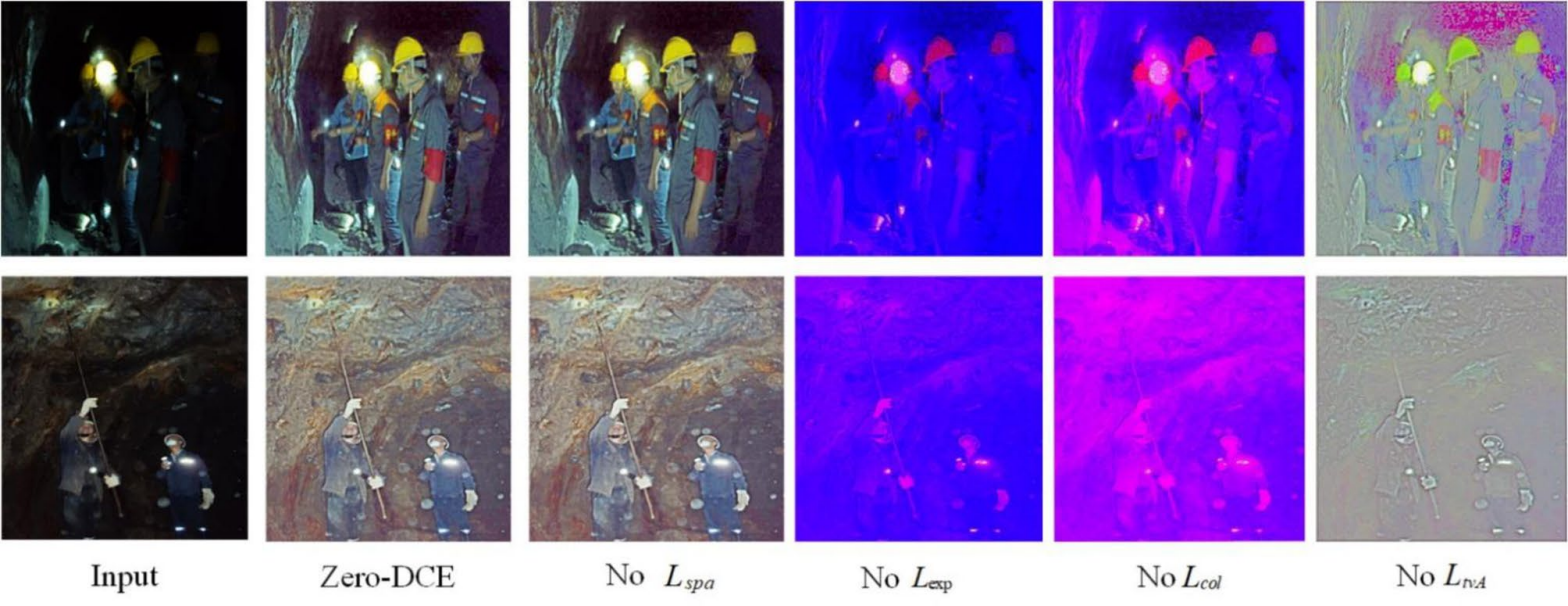
The contribution of each loss to the ablation study, in the absence of spatial consistency loss in the image closest to the model’s initial results, we believe that the loss in the downhole image enhancement contribution is smaller.In summary, we choose the following three types of losses to train our DCE-Net.

Direct visual effects show the impact of each loss function on downhole image enhancement in Fig. 3. To reduce the computational load of the model, we redefine the non-reference loss function.

#### Exposure control loss

To suppress the under-exposure/over-exposure area, we designed an exposure control loss $\:{L}_{exp}$ to control the exposure level. Loss $\:{L}_{exp}$ can be expressed as

1 $$\:{L}_{exp}=\frac{1}{M}{\sum\:}_{k=1}^{M}\left|{Y}_{k}-E\right|$$

Where, $\:\text{M}$ represents the number of non-overlapping local regions with a size of 16 × 16, and $\:\text{Y}$ is the average intensity value of the local region in the enhanced image. We set $\:\text{E}$ to 0.4 empirically.

#### Color constancy loss

The assumption of color constancy in the gray world is followed, that is, the colors in each sensor channel are on average gray across the entire image. Color constancy loss $\:{L}_{col}$ can be expressed as

$$\:{L}_{col}={\sum\:}_{\forall\:\left(p,q\right)\in\:\epsilon\:}{\left({J}^{p}-{J}^{q}\right)}^{2},$$

2 $$\:\epsilon\:=\left\{\left(R,G\right),\left(R,B\right),\left(G,B\right)\right\}$$

Where, $\:{\text{J}}^{\text{p}}$ represents the average intensity value of $\:\text{p}\:$channels in the enhanced image, $\:(\text{p},\text{q})$ represents a pair of channels.

#### Illumination smoothness loss

To maintain the monotone relationship between adjacent pixels, A loss of light smoothness is added to each curve parameter plot A. $\:{L}_{tvA}$ is defined as

$$\:{L}_{tvA}=\:\frac{1}{N}\sum\:_{n=1}^{N}\sum\:_{c\in\:\xi\:}{\left(\left|{\nabla\:}_{x}{A}_{n}^{c}\right|+\left|{\nabla\:}_{y}{A}_{n}^{c}\right|\right)}^{2},$$

3 $$\:\xi\:=\left\{R,G,B\right\}$$

Where N is the number of iterations, $\:{\nabla\:}_{x}$ is the horizontal gradient, and $\:{\nabla\:}_{y}$is the vertical gradient.

#### Total loss

to reduce the calculation amount of the model, we deleted the spatial consistency loss in the original network, and we redefined the complete loss as.

4 $$\:{L}_{total}={L}_{exp}+{{W}_{col}L}_{col}+{{W}_{tvA}L}_{tvA}$$

Where, $\:{\text{W}}_{\text{c}\text{o}\text{l}}$ and $\:{\text{W}}_{\text{t}\text{v}\text{A}}$ are loss weights that used to balance $\:{\text{L}}_{\text{e}\text{x}\text{p}}$, $\:{\text{L}}_{\text{t}\text{v}\text{A}}$ and $\:{\text{L}}_{\text{t}\text{v}\text{A}}$.

### Image dehazing module

The reformulated non-reference loss function to measure the enhanced image quality implicitly and drive the learning of the network is proved to be effective, but the simple function-driven enhancement brings problems such as overexposure, color bias and aggravating blur for downhole images.

To solve the above picture quality problems, the post-processing method of low-light enhancement and deblurring was taken into account in the underground image. AOD-Net was adopted as the backbone of the defogging enhancement module to be explained in Fig. 2- (b), which included five convolutional layers, parallel convolution with filters of different sizes, and the coarse-scale network features were connected with the intermediate layer of the fine-scale network. Finally, multi-scale features are formed by fusing filters of different sizes^33^.

AOD-Net based a reformulated atmospheric scattering model estimates $\:K\left(x\right)$ from input $\:I\left(x\right)$, followed by a clean image generation module that uses $\:K\left(x\right)$ as its input adaptive parameter to estimate $\:J\left(x\right)$. The K-estimation module is a key component of AOD-Net and is responsible for estimating depth and relative haze levels. The model is expressed as follows:

$$\:J\left(x\right)=K\left(x\right)I\left(x\right)-K\left(x\right)+b,$$

5 $$\:K\left(x\right)=\frac{\frac{1}{t\left(x\right)}\left(I\left(x\right)-A\right)+\left(A-b\right)}{I\left(x\right)-1},$$

$$\:t\left(x\right)={e}^{-\beta\:d\left(x\right)}$$

where, $\:I\left(x\right)$ is the fuzzy image after low-light enhancement, $\:J\left(x\right)$ is the scene emissivity(i.e. the ideal “clean image”), $\:b$ is the constant bias of the default value 1, the parameter $\:A$ represents the global atmospheric light, $\:t\left(x\right)$ is the transmission matrix, $\:\beta\:$ is the scattering coefficient of the atmosphere, and $\:d\left(x\right)$ is the distance between the object and the camera.

During the image dehazing stage, we employ Mean Square Error (MSE) as the loss function. The MSE loss directly calculates the pixel-level difference between the generated image and the target image, which can be mathematically expressed as:

6 $$\:{L}_{MSE}=\frac{1}{N}\sum\:_{i=1}^{N}{\left(J\left({x}_{i}\right)-\widehat{j}\left({x}_{i}\right)\right)}^{2}$$

where, $\:\widehat{j}\left(x\right)$ denotes the ground truth image, *N* represents the total number of pixels in the image, and *i* indicates the pixel position index.

## Experiment

**Fig. 4:**
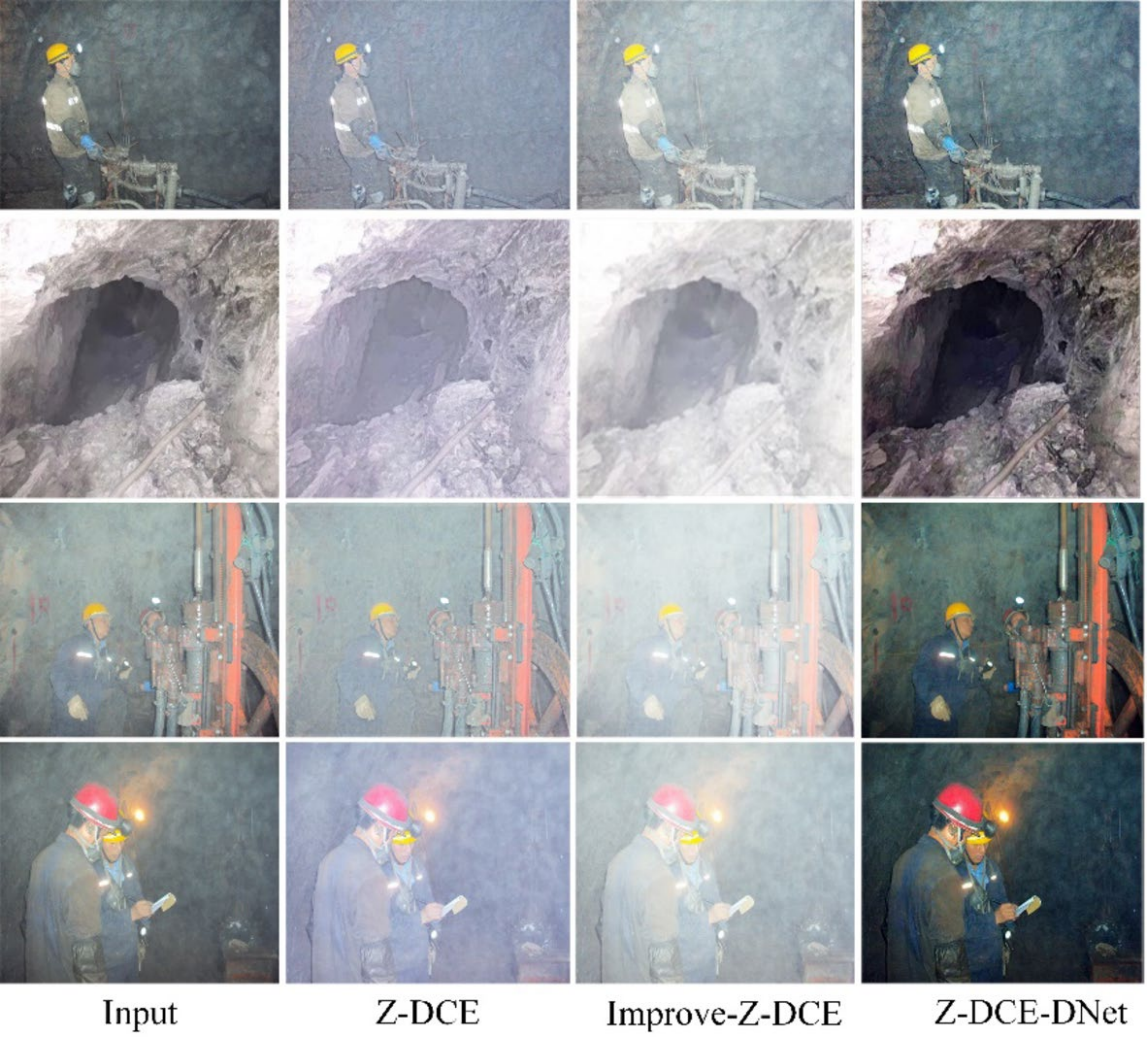
Comparison of Z-DCE-D ablation experiments. our method makes the image clearer for subjective vision.

**Fig. 5:**
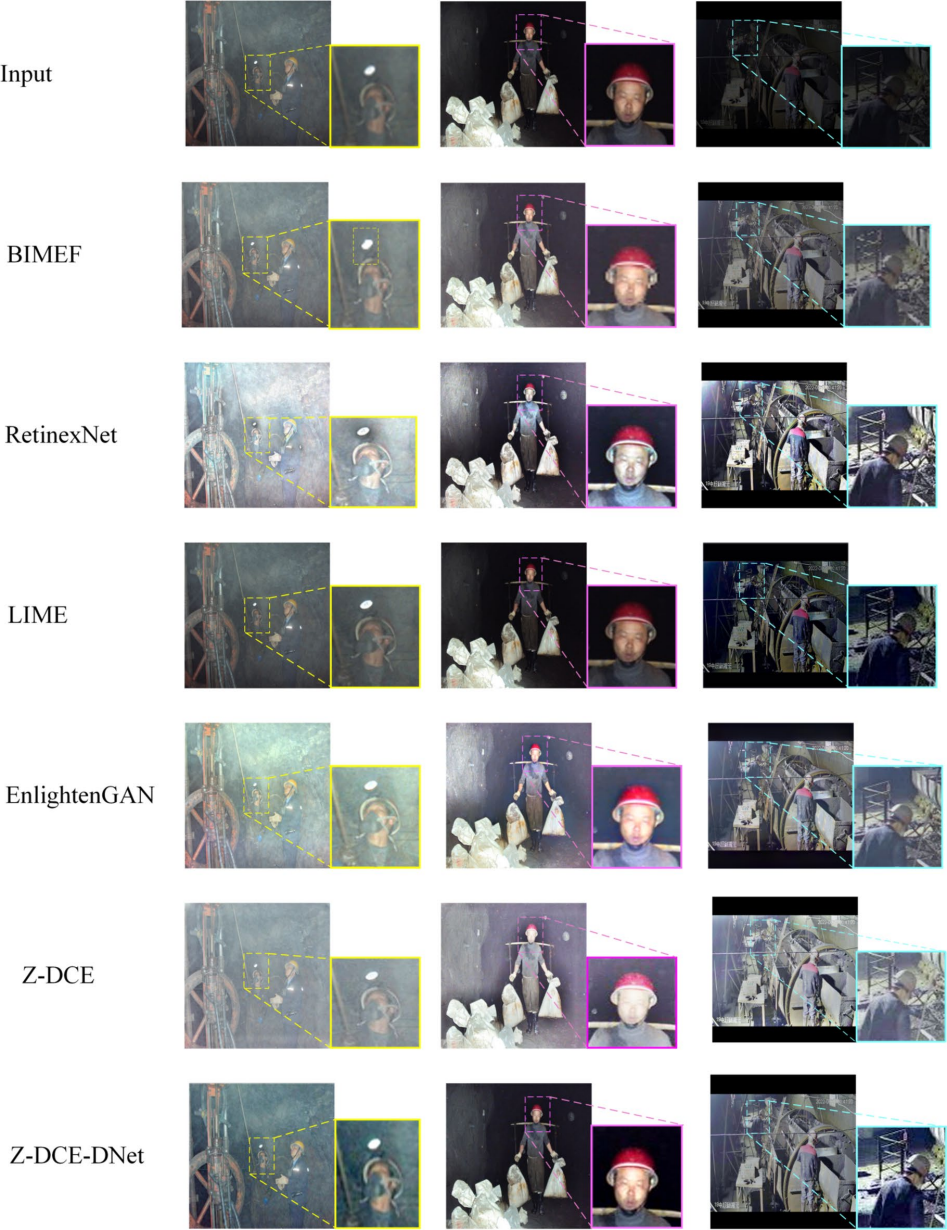
Visual comparison of typical image enhancement. The yellow dotted box in the third row shows that BIMEF algorithm generates part of artifacts while enhancing illumination, and the fourth column shows that LIME algorithm has weak illumination enhancement effect for downhole images.

In this section, the experiment configurations and results using the Z-DCE-DNet for Underground Mine image enhancement are presented. Firstly, the PyTorch deep learning framework was used, running on an NVIDIA GeForce RTX 1650 GPU, applied to underground mine images with a resolution of 512 × 512 for performance measurement. Various algorithms, including object detectors like YOLOv5, YOLOv8, and SSD, were utilized to compare the effectiveness of object grasping in images before and after enhancement. Figures 4 and 5 display visual comparisons of enhanced images. Performance metrics such as PSNR and SSIM values confirm the visual quality of the proposed enhancement technique. The detection results from the object detectors, along with performance metrics, indicate the effectiveness of the proposed method.

### Experiments settings

Z-DCE-DNet was trained on a dataset of 1,647 authentic underground mining images. By incorporating diverse underground scenes into the training set, we fully utilized the advantages of wide dynamic range adjustment. For the IUMD experimental dataset, the images were randomly partitioned into training, validation set and test sets at an 7:2: 1 ratio, with all images standardized to a resolution of 512 × 512.

We realized our framework on Windows 10 system and PyTorch deep learning framework; the GPU is set to NVIDIA GeForce RTX 1650, video memory is set to 4 GiB, batch size is set to 4, and epoch is set to 100. Standard Gaussian functions with zero mean and 0.02 standard deviation are used to initialize the filter weights for each layer. The deviation is initialized to a constant. We use an ADAM optimizer with default parameters and a fixed learning rate of 1e-4 for network tuning. To balance the scale of the losses.

### Ablation study

Ablation experiments based on Zero-DCE networks have been investigated to obtain optimal luminance-enhanced downhole images. A series of experiments demonstrates the methodological research in this paper. We input UMID into the training Z-DCE network to obtain the model Epoch99.pth for verification and testing of downhole images. We randomly verified a set of downhole images shown in Fig. 4. The results obtained by Zero-DCE have a significant brightening effect, but the output images are colored in the visual effect.

We made the following considerations to improve the network performance to solve the color bias problem caused by low light enhancement : (1) Starting from the RGB color channel, we reduced the value proportion of the R channel and redefined the color function inside the Zero-DCE network, (2) For the definition of a group of non-reference loss functions in Formula 4, we re-adjust the size of the weights $\:{\text{W}}_{\text{c}\text{o}\text{l}}$ and $\:{\text{W}}_{\text{t}\text{v}\text{A}}$, respectively set to 5 and 20, to get the results of the output of Improve-Zero-DCE to solve the color bias problem existing in the source code.

Observing the image output in Improve- Zero-DCE, we found that the image after the color correction was too white and the image was blurred. Such an image was not clear and was related to the dust blur present in the underground environment itself. We applied the AOD-Net image deblurring scheme to obtain a cleaner and clearer image. The experiments were verified in Fig. 4, and Z-DCE-DNet showed a superior ability to handle extreme lighting conditions.

The experimental results in Table 1 demonstrate the effectiveness of our proposed Z-DCE-DNet method through comprehensive quantitative evaluation. Compared with baseline methods, Z-DCE-DNet achieves the best performance with PSNR of 13.97 dB and SSIM of 0.643, indicating better preservation of image content and structural information. Furthermore, it obtains the lowest NIQE score of 4.14, suggesting superior perceptual quality of the enhanced images. Although Z-DCE shows better BRISQUE performance, the overall superiority of Z-DCE-DNet across multiple metrics validates its effectiveness for low-light image enhancement.

**Table 1:** Quantitative comparison of image enhancement results in Fig. 4. PSNR (Peak Signal-to-Noise Ratio) and SSIM (Structural similarity index Measure) are full-reference metrics where higher values indicate better quality. PSNR measures the ratio between the maximum possible signal power and noise power, while SSIM assesses structural similarity between images.niqe (Natural image quality Evaluator) and BRISQUE (Blind/Reference-free image Spatial quality Evaluator) are no-reference metrics where lower values indicate better quality. These metrics evaluate perceptual image quality without requiring reference images, which is particularly useful for real-world enhancement tasks.

Method	PNSR↑	SSIM↑	NIQE ↓	BRISQUE↓
Z-DCE	11.36	0.494	6.58	51.44
Improve-Z-DCE	12.51	0.622	6.75	57.45
Z-DCE-DNet	**13.97**	0.643	**4.14**	**62.65**

### Contrast experiments

We compared our method with several advanced methods: including two conventional methods (BIMEF^34^, LIME^35^, a CNN-based method RetinexNet^15^, and a GAN-based method EnlightenGAN^20^).

Three downhole images from each scene were randomly selected for verification. Yellow, pink and blue block diagrams were used to compare the effects of each algorithm model. As shown in Fig. 5, the BIMEF model can perform color processing well in low-light image enhancement, while Retinex-Net and EnlightenGAN two methods have a huge color bias problem after enhancement. Compared with the results of multiple experimental data, our method can achieve better visual effects for image processing of each scene in the mines.

A quantitative comparison of various enhancement methods, encompassing both objective metrics and architectural characteristics in Table 2. The experimental results reveal notable performance variations across different assessment criteria.

Quantitatively, while Z-DCE achieves optimal PSNR performance (32.45) and LIME demonstrates superior SSIM metrics (0.8870), our proposed Z-DCE-DNet exhibits balanced performance across all evaluation metrics. Particularly noteworthy is its exceptional performance in no-reference metrics, achieving the optimal NIQE score (6.22) and BRISQUE value (44.89), which are crucial for real-world mining applications where reference images are unavailable.

From an architectural perspective, the methods exhibit distinct computational characteristics. Conventional approaches such as BIMEF and LIME employ non-neural methodologies with iterative optimization processes. In contrast, deep learning-based methods demonstrate varying degrees of architectural complexity. EnlightenGAN incorporates a sophisticated GAN framework with U-Net architecture and self-attention mechanisms, necessitating substantial computational resources. RetinexNet implements a multi-stage network architecture with deep reconstruction components, resulting in increased memory requirements.

Our proposed Z-DCE-DNet distinguishes itself through its efficient architectural design, combining DCE-Net and AOD-Net in a streamlined framework. This approach achieves competitive enhancement performance while maintaining minimal computational overhead, as evidenced by the ‘Network Architecture’ and ‘Parameters’ specifications in Table 2. The lightweight design principles employed in Z-DCE-DNet make it particularly advantageous for deployment in practical mining environments where computational efficiency is paramount.

**Table 2:** Comprehensive comparison of image enhancement methods in terms of quantitative metrics and computational characteristics. The best and second-best results are marked in red and Blue, respectively.

Method	PNSR↑	SSIM↑	NIQE ↓	BRISQUE↓	Network Architecture	Parameters
BIMEF^34^	31.47	0.8656	6.92	47.47	Multi-exposure fusion	Multiple exposure processing steps
LIME^35^	32.42	0.8870	6.67	47.52	Initial map estimation	Iterative optimization process
RetinexNet^15^	29.57	0.8556	6.77	46.92	Decomposition network	Multi-stage network with deep reconstruction
EnlightenGAN^20^	31.21	0.8514	6.60	46.09	U-Net、Self-attention modules	Complex GAN architecture requiring significant memory
Z-DCE^26^	32.45	0.8595	6.92	47.52	DCE-Net	Lightweight design with minimal parameters
**Z-DCE-DNet**	31.93	0.8700	6.22	44.89	DCE-Net、AOD-Net	Lightweight design with minimal parameters

### Object detection experiment

**Fig. 6:**
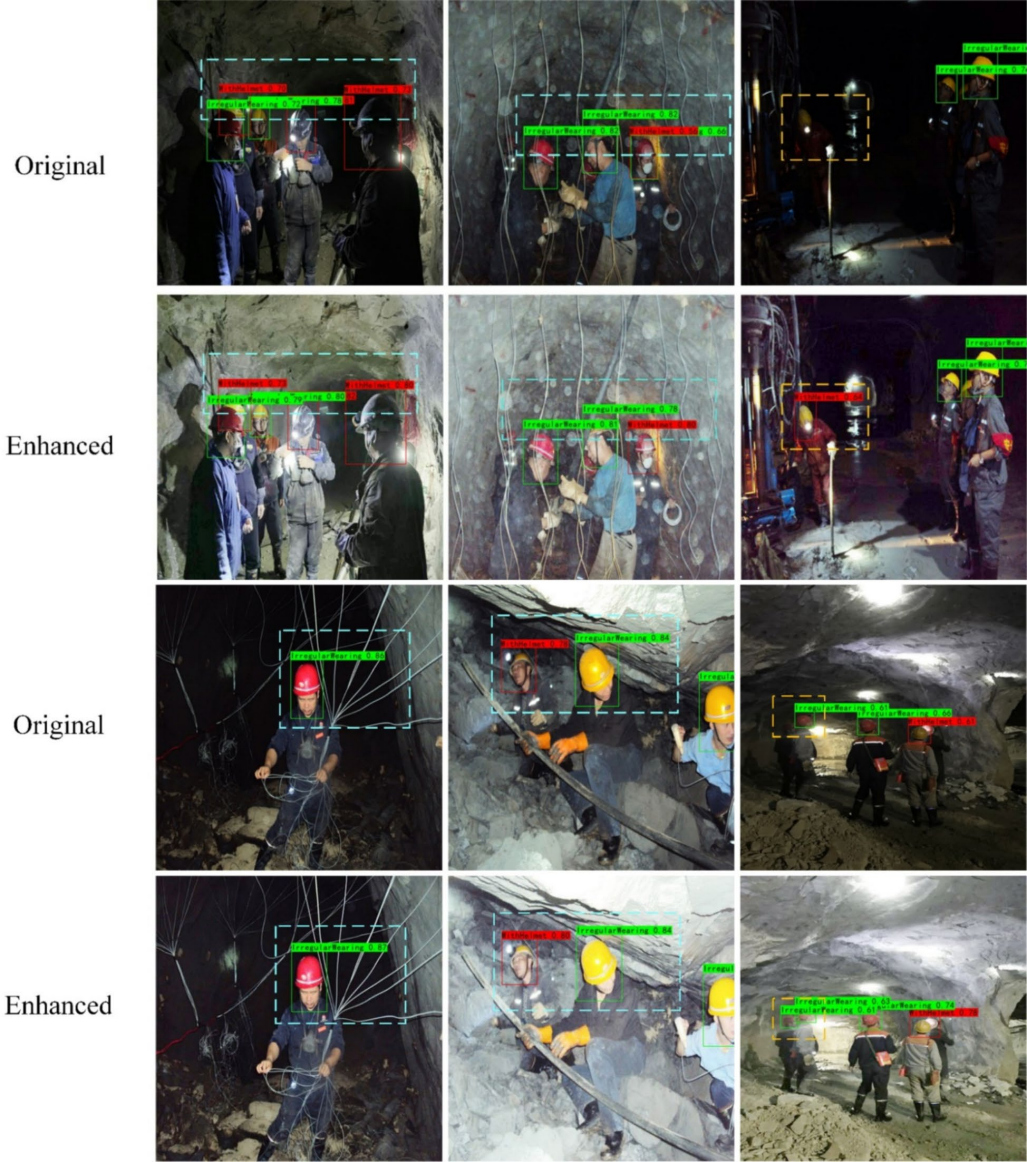
Comparison of safety helmet detection results before and after subsurface image enhancement. The blue box shows that the detection confidence of the safety helmet detection model after enhancement is significantly improved, and the yellow box shows the accurate detection of the enhanced image after missing the original image. The result proves that downhole image enhancement can enhance the feature of the target object, which is conducive to the monitoring of downhole vision equipment.

To verify the subsequent surveillance effectiveness of the enhanced images in underground mine video surveillance, further target detection was conducted, and a trained underground safety helmet detection model^36^ was adopted to compare the original image detection with the enhanced image detection using our method. The results are shown in Fig. 6. Our method yielded superior visual effects, enhancing the visibility of the target safety helmets more prominent. Furthermore, upon comparing the two sets of experimental charts, it can be observed that the enhanced images exhibited superior detection accuracy in the results of the same safety helmet detector. Especially in terms of detector confidence and precise detection, the enhanced images demonstrated a significant improvement.

Based on the above experimental content, we further investigated the impact of enhanced images on target detection in underground mine. The experimental content is as follows: We utilized enhanced images in YOLOv5, YOLOv8, and SSD target detectors for model training and compared the Precision, Recall, mAP, and F1-score of each model.

#### Performance metrics

Average Precision (AP), mean Average Precision (mAP), and harmonic mean (F1) are commonly used in target detection as standards for evaluating the accuracy performance of detection models. The calculation formulas are shown in the following equation:

6 $$\:AP={\int\:}_{0}^{1}PdR$$

7 $$\:\text{m}\text{A}\text{P}=\frac{\sum\:_{\text{i}=1}^{\text{k}}\text{A}{\text{P}}_{\text{i}}}{\text{k}}$$

8 $$\:\text{F}1=2\times\:\frac{\text{P}\times\:\text{R}}{\text{P}+\text{R}}$$

In the formula, AP represents the average precision for a single class, mAP is the average of all class AP values, F1 is the harmonic mean of P and R, where P is precision, R is recall, and k is the number of detected categories; the calculation formulas for P and R are as follows:

9 $$\:P=\frac{TP}{TP+FP}$$

10 $$\:\text{R}=\frac{\text{T}\text{P}}{{\text{N}}_{\text{G}\text{T}}}$$

In the formula, TP represents the number of true positive prediction boxes, FP represents the number of false positive prediction boxes, $\:{\text{N}}_{\text{G}\text{T}}$ represents the count of ground truth values.

#### Discussion and analysis

**Fig. 7:**
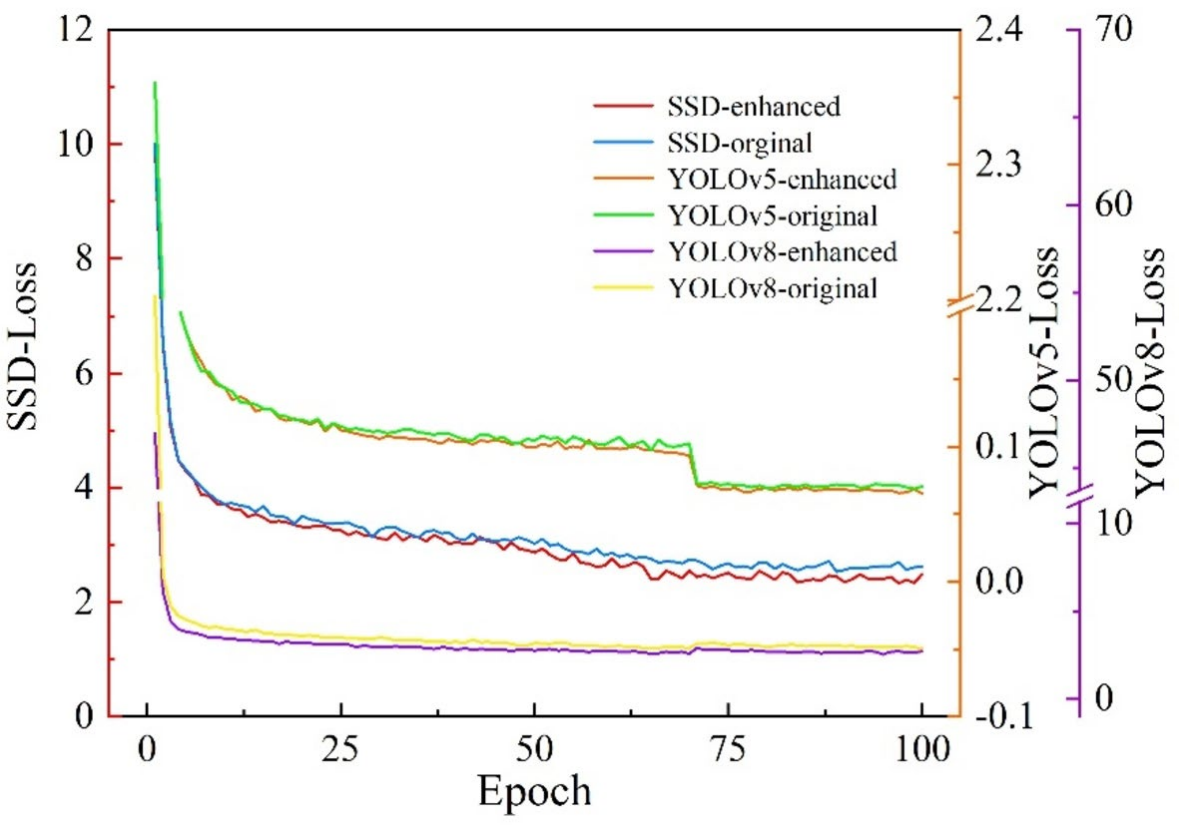
Comparison of Loss curves of each model training. The enhanced image is more conducive to the convergence of the loss curve of the target detection model.

The influence of enhanced images on target detection models in underground mines was further investigated based on the above experimental content. The enhanced images were used to train the YOLO series and SSD target detection models, and the performance results of each model were compared, as shown in Table 3. The experiment proves that the enhanced image object features are more significant, which is beneficial to the work of vision equipment and has a positive effect on the development of vision software. The comparison of the loss curves of the model training is shown in Fig. 7.

**Table 3:** Performance pairs of training results of images before and after enhancement on target detection model. Red indicates better results.

Model	Precision↑	Recall↑	AP (Average Precision)↑	F1↑
YOLOv5(original)	93.27	81.98	94.28	0.88
YOLOv5(enhanced)	94.79	82.91	94.88	0.88
YOLOv8(original)	98.02	89.19	97.91	0.93
YOLOv8(enhanced)	95.69	94.87	97.92	0.95
SSD(original)	93.07	80.34	88.63	0.86
SSD(enhanced)	93.88	82.88	91.66	0.88

While training models, we often analyze the model by examining the loss function curve of the target detector to optimize the training parameters for a more robust model with improved generalization capabilities. For the safety helmet detector based on enhanced underground images, training was conducted for 100 epochs, and the loss curves of each model are shown in Fig. 7. Initially, all models converge, but due to differences in the complexity of their network layers, they exhibit varying loss values. Secondly, among all models, enhanced images demonstrate better convergence performance.

## Conclusion

This thesis discusses the prior knowledge of deep convolutional neural networks. We investigate the Z-DCE-DNet for the restoration of underground images. We combine low-light image enhancement with post-processing image denoising. The pixel range of the image is adjusted by improving the network parameters and the non-reference loss function. After a wealth of experiments, it is proved that our method can not only achieve certain results in visual effects, but also enhance the features of the detected objects in the subsequent target detection, and is more conducive to the training of the target detection model.

## Data Availability

The datasets used and/or analysed during the current study available from the corresponding author on reasonable request.
